# Lightweight High-Performance Polymer Composite for Automotive Applications

**DOI:** 10.3390/polym11020326

**Published:** 2019-02-13

**Authors:** Valentina Volpe, Sofia Lanzillo, Giovanni Affinita, Beniamino Villacci, Innocenzo Macchiarolo, Roberto Pantani

**Affiliations:** 1 Department of Industrial Engineering, University of Salerno, via Giovanni Paolo II 132, Fisciano, 84084 Salerno, Italy; rpantani@unisa.it; 2 SAPA s.r.l. Via Appia Est, 1 82011 Arpaia, 82011 Benevento, Italy; sofia.lanzillo@sapagroup.it (S.L.); giovanni.affinita@sapagroup.it (G.A.); beniamino.villacci@sapagroup.it (B.V.); innocenzo.macchiarolo@sapagroup.it (I.M.)

**Keywords:** microcellular injection molding, polyamide 66, morphology, ANOVA

## Abstract

The automotive industry needs to produce plastic products with high dimensional accuracy and reduced weight, and this need drives the research toward less conventional industrial processes. The material that was adopted in this work is a glass-fiber-reinforced polyamide 66 (PA66), a material of great interest for the automotive industry because of its excellent properties, although being limited in application because of its relatively high cost. In order to reduce the cost of the produced parts, still preserving the main properties of the material, the possibility of applying microcellular injection molding process was explored in this work. In particular, the influence of the main processing parameters on morphology and performance of PA66 + 30% glass-fiber foamed parts was investigated. An analysis of variance (ANOVA) was employed to identify the significant factors that influence the morphology of the molded parts. According to ANOVA results, in order to obtain homogeneous foamed parts with good mechanical properties, an injection temperature of 300 °C, a high gas injection pressure, and a large thickness of the parts should be adopted.

## 1. Introduction

One of the most common methods for the production of plastic parts in automotive industry is the conventional injection molding process. However, the need of the automotive industry to produce plastic products with high dimensional accuracy and reduced weight drives the research towards less conventional industrial processes. Among these, a promising method is the microcellular injection molding process, which allows for the production of complex, thick parts with a cellular core and a solid skin [1]. The key insight of microcellular injection molding, as developed and commercialized by Trexel Co. Ltd. as the Mucell process [2], is the application of a supercritical fluid [3]. In particular, the expansion of a physical blowing agent in the polymer melt allows for the formation of a foamed core that decreases the part weight using less raw material [4,5,6,7]. The main advantages of microcellular injection molding process are the reduction of injection pressure and cycle time, 10% to 30% saving in raw material, and the improvement of the dimensional stability of the molded parts [4,8,9]. Furthermore, the morphology of foamed parts can, in principle, be arranged via controlling the processing parameters, in order to generate different microcellular foam polymers with desired properties, which are suitable for use in different industrial areas [10,11]. Chen et al. in 2004 [12] studied the effects of several factors, like including molding parameters, part thickness, and foaming agent content, on molding characteristics and the mechanical strength of injection molded foaming polypropylene parts. They assessed that the part thickness is a dominant factor to determine the degree of foaming: thicker parts allows for higher specific weight reduction. It has to be mentioned that foaming induces weight reduction, but it also causes a loss of mechanical properties. In 2014, Guo et al. [10] investigated the effects of processing parameters in microcellular injection molding in order to reduce weight and improve the dimensional accuracy of plastic products for automotive industry. On the basis of fractional factorial design, they found that initial gas concentration and melt temperature are the most significant parameters in microcellular injection molding.

The most common materials that are adopted in microcellular injection molding process are commodity plastics, such as polypropylene and polystyrene [13]. However, the need of the automotive industry to have light-weight composites with good stiffness and strength in the molded part led to the search for fillers to reinforce these materials. Among the different fillers, glass fibers (GF) are the most commonly adopted because of their excellent balance between low cost, high stiffness and strength, high chemical resistance, and insulating properties [14,15,16]. The use of glass fibers induces an increase of part weight, which can be limited by foaming, thus making this process even more interesting for composites. It has to be mentioned that engineering plastics, which are high performance thermoplastic materials with better mechanical and thermal properties than commodity plastics, represent a much less studied class of materials for microcellular injection molding. In 2015, Gómez-Monterde et al. [17] conducted the morphology characterization of ABS foams that were obtained by injection molding, as well as an analysis of the effects of gas content and density reduction on the mechanical behavior. They produced structural foams with a solid skin and a foamed core divided in two parts: a nucleus, with bigger cells and irregular cell distribution due to a higher expansion rate and bubble coalescence, and a microcellular zone between the nucleus and the skin layer, with more homogenous cell structure. Their results showed a gradual decrease in the mechanical properties with decreasing apparent density. Recently, Luo and his research group studied the effects of several processing parameters on the cell morphology and the properties of a series of microcellular polyetherimide (PEI) foams that were prepared by microcellular injection molding with supercritical nitrogen as foaming agent [13,18]. Another engineering plastic adopted for microcellular injection molding is polyamide 6 [19,20]. This material is extremely important for the automotive industry, for under-the-hood components. With the aim of reducing part weight, the application of foam injection molding to these plastics becomes a strategic factor. Yuan and Turng examined the microstructures of microcells, nanoclay, and crystals in polyamide 6 microcellular nanocomposites and the corresponding influence of nanoclay, crystallites and microcells on part properties [21,22,23]. They concluded that, for polyamide 6 and its composites, microcellular injection molded plastics generally have lower tensile strength and impact strength when compared to their solid counterparts. Liu et al. [24] investigated the cell structure, mechanical properties, crystallization behavior, and dielectric property of microcellular PPS foams. The results showed a decline of mechanical properties and dielectric constant with the decrease of the relative density of the foamed materials. Ma et al. [25] in 2016 investigated the effects of processing conditions on the foaming behaviors and the morphology of the microcellular foamed PPS/GF (glass fibers) composites. They found that the presence of GF can induce the PPS to form a particular transcrystalline structure around the glass fibers by generating a heterogeneous cell nucleation.

In this study, the microcellular injection molding was carried out by using glass-fiber-reinforced polyamide 66, a high performance material that is of great interest for the automotive industry but still scarcely studied for this process. With respect to polyamide 6, polyamide 66 is more expensive but it presents a better thermal resistance. This characteristic makes this plastic extremely important for under-the-hood components. With the aim of reducing part weight, the application of foam injection molding to this plastics can be extremely interesting for industrial applications. The influence of key processing parameters, such as injection temperature, gas injection pressure, and part thickness on the morphology of PA66 + 30% glass fiber foamed parts, on their density reduction, thermal, and mechanical performances was investigated. Furthermore, in order to identify the most significant factors that influence the morphology of the molded parts, an analysis of variance (ANOVA) was applied.

## 2. Materials and Methods

In this work, a polyamide 66 (PA66) HERAMID A NER GF030/1K reinforced with glass fiber at 30% by weight produced by Radici group Plastics (Radici Partecipazioni SpA, Bergamo, Italy) was adopted. This material has a melting temperature of 260 °C and it is conventionally used in production engine covers and other automotive parts because of its good thermal and mechanical properties. The glass fiber length was measured to be about 500 micron.

### 2.1. Experimental Protocol

**Figure 1:**
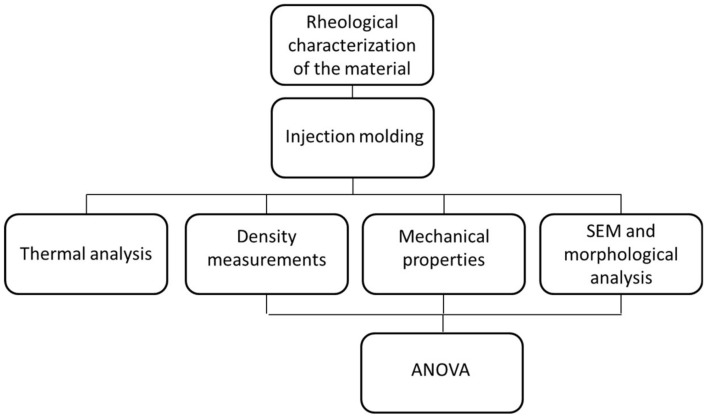
Flow diagram of the experimental protocol.

Figure 1 schematically shows a flow diagram of the experimental protocol.

### 2.2. Rheology

The viscosity of the PA66 reinforced with glass fiber at 30% by weight was measured at 280 °C and at 300 °C by means of a capillary rheometer RH7 Flowmaster Bohlin Instruments (Malvern Panalytical Ltd., Malvern, UK, Diameter 1 mm, length 10 mm), in the range of shear rates from 5/s to 5000/s. A regression analysis was then conducted in order to find the parameters of the Cross-WLF model [26]. In particular, the dependence of shear viscosity upon shear rate was assumed to agree with the Cross model:

(1) $$\eta =\frac{\eta_{0}}{1+{\left(\frac{\eta_{0}\dot{\gamma }}{\tau^{*}}\right)}^{1-n}}\;,$$

where *η* is the melt viscosity, *η*_0_ is the zero shear viscosity, $\dot{\gamma }$ is the shear rate, $\tau^{*}$ is the critical stress level at the transition to shear thinning, and n is the power law index. The zero shear viscosity was assumed to be given by the WLF equation:

(2) $$\eta_{0}=D_{1}exp [-\frac{A_{1}\left(T-T^{*}\right)}{A_{2}+\left(T-T^{*}\right)}]\;,$$

where T is the temperature, $T^{*}$ is the glass transition temperature, and $D_{1}$, $A_{1}$, $A_{2}$ are data-fitted coefficients. The parameters appearing in Equations (1) and (2) were found by minimizing the error between the predictions that were obtained by equations and the experimental results of shear viscosity. The description was very good, so that the predictions were essentially coincident with experimental data at all temperatures and for all of the shear rates investigated.

**Figure 2:**
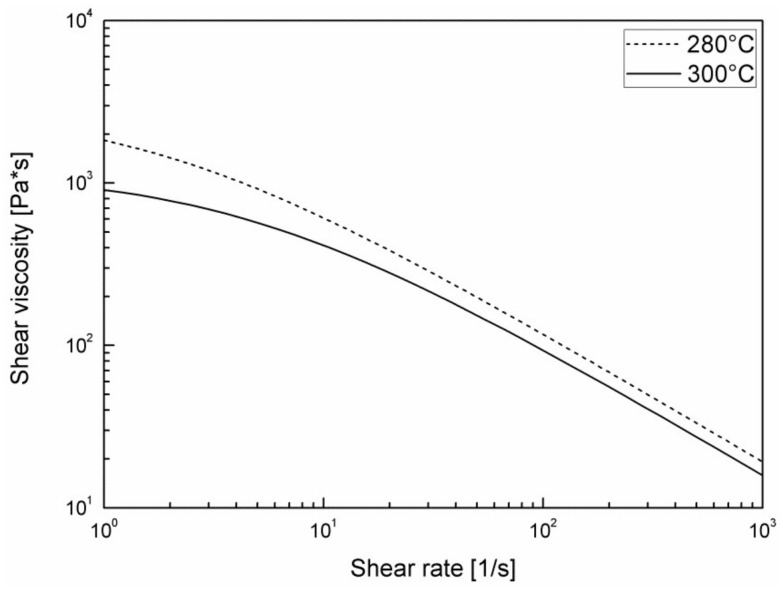
Shear viscosity of the nylon 66 adopted in this work.

In Figure 2, it is possible to observe the so-called flow curve of the material, namely the dependence of shear viscosity on shear rate. The parameters adopted to describe the measurements by the Cross-WLF Model are shown in Table 1.

### 2.3. Injection Molding

**Figure 3:**
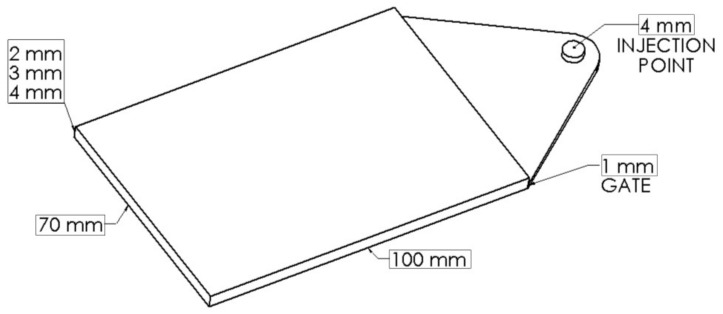
Cavity geometry.

PA66 with glass fibers was processed by an injection molding machine (70 ton Negri Bossi, screw diameter 25 mm, L/D = 22) equipped with gas injectors that were connected to a volumetric pump and a screw with a mixing section. The mold was equipped with a hot runner with a gate valve. Nitrogen in supercritical state was adopted as physical blowing agent for the foam injection molding process. The geometry of the cavity is reported in Figure 3.

In this work, samples with three different thicknesses (2, 3, and 4 mm) were produced. The material was processed by setting two different injection temperatures, 280 and 300 °C. For each injection temperature, two gas injection pressures were adopted, corresponding to two amounts of gas (in grams) that were injected in the polymer melt. In particular, the volumetric pump allowed the monitoring of the amount of gas injected into the cylinder during the batching step. The number of moles injected and the corresponding amount in grams were calculated on the basis of the values of pressures, temperature, and volumes of nitrogen that are present in the pump before and after the injection. The experimental conditions are summarized in Table 2.

It has to be mentioned that, in this work, a low-pressure variant of the foam injection molding was adopted. This means that for each condition a suitable short shot was produced in such a way that, after expansion, the polymer completely filled the cavity and a complete part was produced. As a consequence, the amount of material injected changes for each molding condition.

### 2.4. Density Measurements

Density measurements were performed at 25 °C by weighing the samples, deprived of the part before the gate (which contains the injection point), immersed in water on the basis of Archimedes’ principle. The density reduction is expressed as:

(3) $$Density\;reduction=\frac{\rho_{0}-\rho_{f}}{\rho_{0}}\times 100\;,$$

where $\rho_{0}$ is the density of an unfoamed sample and $\rho_{f}$ the density of the foamed sample.

### 2.5. Mechanical Properties

In order to correlate the mechanical properties to the morphology of the foamed parts, flexural tests were carried out by means of a universal testing machine ATSFAAR TC1000 (ATS FAAR Industries srl, Milano, Italy). In particular, a load cell of 2 kN and a rate of crosshead nose of 1 mm/min (according to ASTM D 790-03) were adopted. The tests were conducted in three-point bending mode on 15 mm wide specimens that were cut at a distance of 45 mm from the gate. In order to give a true comparison between the samples with different densities, the flexural modulus that was obtained by the stress-strain curves was normalized according to Equation (4) [11,27], where E and ρ are, respectively, the modulus and the density of the foamed sample, while $E_{0}$ and $\rho_{0}$ are referred to an unfoamed sample.

(4) $$Normalized\;modulus=\frac{E}{E_{0}}\left(\frac{\rho_{0}}{\rho }\right)\;,$$

Mechanical properties are closely correlated to the sample morphology. From the literature, it is known that the flexural modulus is essentially determined by the unfoamed skin layer, which is mostly solicited in this kind of test [7,11]. However, this normalization does not take in account the effect that is produced by the non-uniform density across the foam section due to the compact skin and the foamed core. The approach that was proposed by Gonzalez [28] assimilates the molded part to a sandwich structure, with a skin layer having the modulus of the matrix and a core section of uniform modulus, by assuming that the transition zone is much smaller than the core layer. According to this theory, the average normalized flexural modulus can be given by:

(5) $$\frac{E_{sf}}{E_{m}}=1-{\left(\frac{\delta_{c}}{\delta_{f}}\right)}^{3}+{\left(\frac{\delta_{c}}{\delta_{f}}\right)}^{3}{\left(1-\frac{\delta_{f}}{\delta_{c}}f\right)}^{2}\;,$$

where $E_{sf}$ is the average flexural modulus measured, $E_{m}$ is modulus of matrix, $\delta_{c}$ is thickness of core part, and $\delta_{f}$ is thickness of the structural foam. The void volume fraction f can be calculated as:

(6) $$f=\left(1-\frac{\rho_{c}}{\rho_{m}}\right)\frac{\delta_{c}}{\delta_{f}}\;,$$

where $\rho_{c}$ and $\rho_{m}$ are the density of the core and the density of the matrix, respectively.

### 2.6. Thermal Properties

Heat Deflection Temperature tests (HDT) were conducted on specimens cut at a distance of 45 mm from the gate. The tests were performed in air at a heating rate of 2 °C/min in a range temperature of 25–250 °C according to ISO 75—Method A (1.8 MPa).

### 2.7. Scanning Electron Microscope (SEM)

The samples were sectioned at 45 mm from the gate and analyzed by a LEO-EVO 50 Scanning electron microscopy (SEM) (Carl Zeiss AG, Oberkochen, Germany) in order to observe the effect of the processing parameters on cell morphology. Before the observation, the samples were coated with a 5 nm gold layer to prevent charging during SEM analysis. A software for image analysis allowed for obtaining cell density, cell size distribution, and skin thickness from the SEM images. Cell density was evaluated as:

(7) Cell density=nA32 ,

where n is the number of bubbles in the micrograph of area A in cm^2^. At least two samples for each condition were analyzed. The interconnected cells were counted individually if a boundary was evident: when a cell was clearly formed by two or more different cells, each cell was counted; when instead, it was not possible to detect the single cells constituting a larger, non-spherical one, only one cell was counted.

### 2.8. Analysis of Variance (ANOVA)

The multiplicity of the analyzed parameters allowed for employing an analysis of variance to identify the significant factors that influence the morphology of the molded parts.

One of the most common statistical methods adopted to estimate the contributions of each parameter is the analysis of variance (ANOVA), which can be used to find the most influencing parameters on a given response.

The sum of squares (SS), which measures of the total variability of the observed data, is defined as:

(8) $$CSS=\frac{\sum_{i=0}^{n}X_{a}^{2}}{n}-\frac{T^{2}}{N}\;,$$

where *a* is the factor, *Xa* is the sum of the observed data at factor *a*, *T* is the sum of all of the data, and *N* is the total number of data points. The degree of freedom is the number of levels of each factor minus one. The mean square is defined as the ratio between the sum of square and the degree of freedom. The ratio between the mean square factor and the mean square error represents the F-value. The percentage contribution can be calculated as:

(9) $$Percentage\;contribution=\frac{SS_{a}}{SS_{T}}\times 100\;,$$

where *SS_a_* is the sum of squares of factor a and *SS_T_* is the total sum of squares [29]. For the significance level of the analysis, the probability value, *p*-value, was considered. In particular, a *p*-value of 0.05 was chosen (as normally done for experimental analysis). This indicates that, if the *p*-value is less than or equal to 0.05, a relationship exists between the dependent and the independent variable. Otherwise (i.e., if the *p*-value is greater than 0.05), there are no statistically significant differences between group means, as determined by ANOVA.

In this work, an analysis of variance concerned the dependence of the main results, i.e., part density, normalized flexural modulus, and cell density in the core, on three fundamental factors, injection temperature, part thickness, and gas injection pressure, was implemented.

## 3. Results

**Figure 4:**
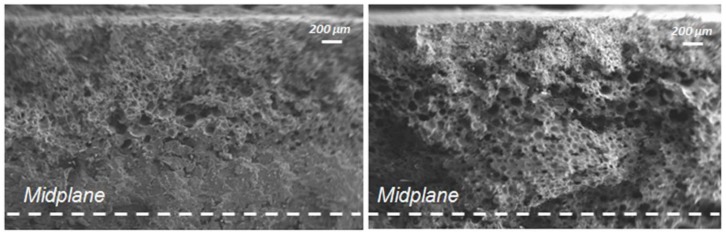
Half-section of samples molded with injection temperature of 280 °C (**a**) and 300 °C (**b**). Sample thickness 3 mm.

A preliminary study was conducted in order to investigate the effect of the injection temperature on the samples morphology. Figure 4 shows a comparison between the cross-section of the samples that were molded with injection temperature of 280 and 300 °C.

In Figure 4, it is possible to observe a non-homogeneous morphology of the sample at 280 °C, with a well foamed transition zone (zone between the external skin and the core) and a completely unfoamed core layer. Vice versa, the sample molded with injection temperature 300 °C showed almost the same transition zone and a well foamed core layer, with a homogeneous cell distribution. This difference can be attributed to the higher viscosity of the thermoplastic material at 280 °C, especially at low shear rates, which does not permit good foaming during the latest stage of the process [30]. For this reason, our research focused on samples that were obtained by setting the injection temperature at 300 °C.

Parts with three different thicknesses were produced by foam injection molding by setting the injection temperature at 300 °C and using two gas injection pressures, corresponding to different gas amounts. Table 3 reports the ratio between the amount of gas in grams and the injection volume for each part of thickness corresponding to the gas injection pressures that were adopted in this work.

**Figure 5:**
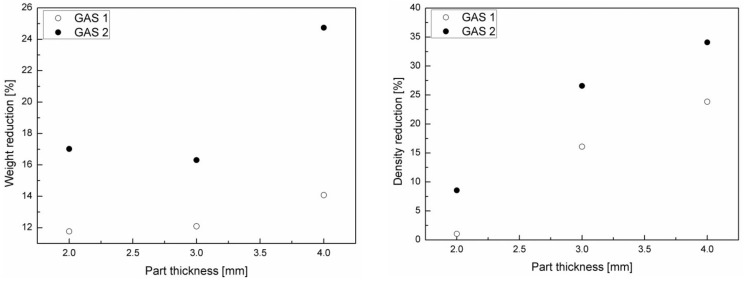
Weight reduction (**a**) and density reduction (**b**) for the three different part thicknesses.

Figure 5 shows weight reduction and density reduction for the three different part thicknesses.

As it can be noticed from the graphs, weight and density reduction increase with the gas injection pressure and with the part thickness. In particular, samples with a part thickness of 4 mm foamed with the highest gas injection pressure achieved a weight reduction of 25% and a density reduction corresponding to 34%. The weight reduction is correlated to the density reduction by the equation:

(10) $$\frac{M_{0}-M}{M_{0}}=\frac{V}{V_{0}}\left(\frac{\rho_{0}-\rho }{\rho_{0}}\right)+1-\frac{V}{V_{0}}\;,$$

The volume of the foamed samples is different from the volume of the corresponding compact samples because, as specified above, they were obtained with different molding conditions in order to obtain—in all cases—a fully foamed sample. This gives rise to different volume shrinkage for each case [31].

### 3.1. Microstructure

**Figure 6:**
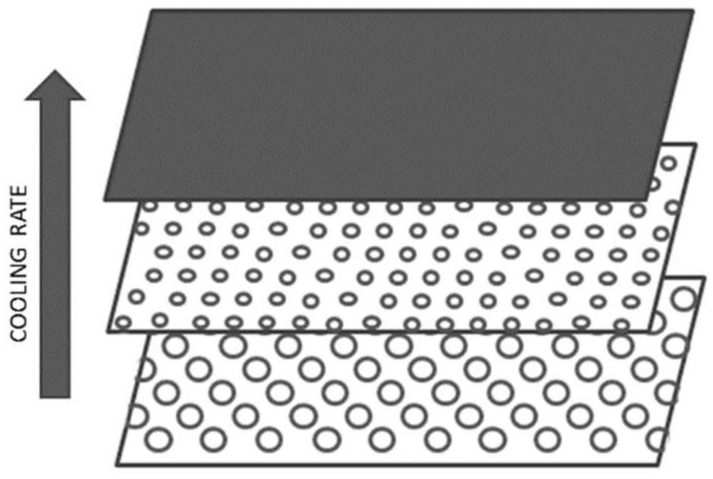
Schematic microstructure of a foamed part.

In order to evaluate the microstructure of the foamed components in the different analyzed conditions, a morphological analysis by scanning electron microscopy was carried out. The microstructure of the foamed components presents three layers: external compact skin, transition zone, and foamed core. In particular, during the injection phase, the material solidifies as soon as it comes into contact with the mold walls, quickly forming a layer of compact skin, while the material that subsequently enters the cavity is subjected to a lower cooling rate that allows foaming [16]. The decreasing cooling rate profile from the mold walls to the middle of the sample allowed for the formation of layers with different foaming degrees (Figure 6).

**Figure 7:**
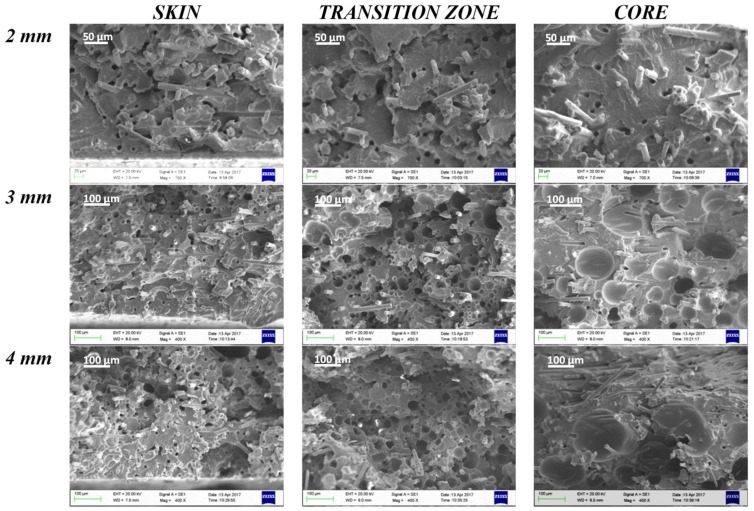
Scanning electron microscopy (SEM) images of skin, transition zone, and core layer of the samples with gas injection pressure 70 bar (GAS 1) at the three analyzed thicknesses.

**Figure 8:**
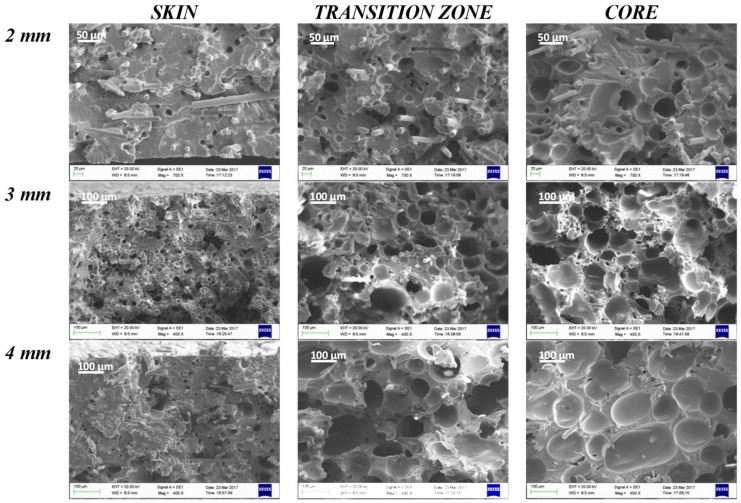
Scanning electron microscopy (SEM) images of skin, transition zone, and core layer of samples that were obtained with gas injection pressure 120 bar (GAS 2) at the three analyzed thicknesses.

In Figure 7 and Figure 8, SEM images of the skin, transition zone, and core layer of the samples (thickness 2, 3, and 4 mm) molded with gas injection pressure 70 bar (GAS 1) and 120 bar (GAS 2), respectively, are shown. In all cases, except the 2 mm GAS 1 sample for which a very low foaming degree is reached, the images clearly show larger cells in the core region with respect to the transition zone, which is in agreement with the scheme reported in Figure 6.

**Figure 9:**
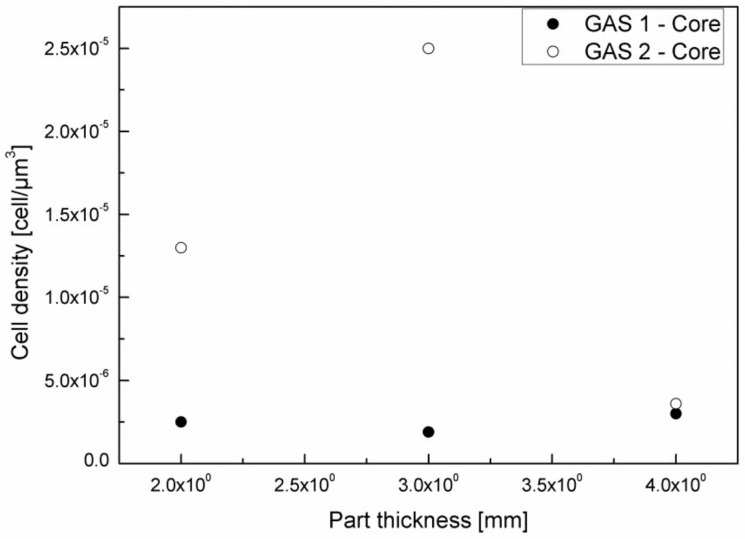
Cell density in the core layer of samples foamed at 70 bar and 120 bar.

From the SEM images, a remarkable improvement in morphology passing from a gas injection pressure of 70 bar (GAS 1) to 120 bar (GAS 2) can be observed. In fact, although GAS 1 samples present a fairly foamed transition zone, the skin thickness is significant and the core layer is substantially compact or is composed of large size cells, at least in the case of thicker samples. Figure 9 reports the cell density in the core layer of the GAS 1 and GAS 2 samples.

Figure 9 clearly shows the substantial difference in cell density between the core layer of GAS 1 and GAS 2 samples. The GAS 1 sample, in fact, presents a cell density that is almost constant with the part thickness and of one order of magnitude lower than the cell density that was obtained for the thicknesses 2 mm and 3 mm of the GAS 2 sample. Thicker samples (4 mm), instead, do not show significant differences in cell density between the GAS 1 and GAS 2 samples, probably because the larger thickness involves a lower cooling rate in the core layer, which leads to cell coalescence.

By analyzing in detail the morphology of the GAS 2 samples, it is possible to note that the skin layer thickness decreases (Table 4) on increasing the sample thickness. This happens because the thicker parts are subjected to a much lower cooling rate than thinner ones. The fast cooling of the thinner GAS 2 samples (2 mm) involves the formation of a compact skin of approximately 30% of the part thickness. In the case of GAS 1 samples, the skin thickness does not show a clear trend with the part thickness, as instead observed for GAS 2.

**Figure 10:**
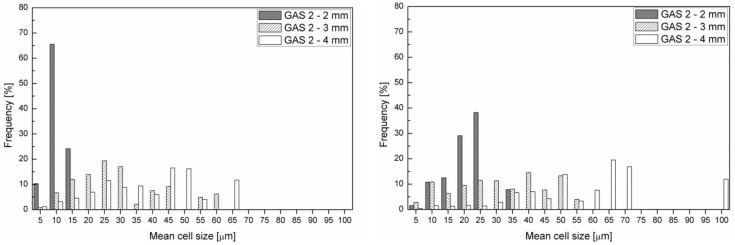
Cell size distribution in the transition zone (**a**) and core (**b**) of samples foamed at 120 bar.

Figure 10 shows the cell size distribution in the transition zone and core of the GAS 2 samples. It is possible to observe that the cell size distribution in the core layer (Figure 10b) is similar to that of the transition zone (Figure 10a), especially in the case of 3 mm and 4 mm thick samples. This indicates homogeneous foaming. Furthermore, by observing Figure 10, it can be concluded that the mean cell size increases with the part thickness. In the case of core layer, the mean cell size increases until it reaches remarkably high values, which can be observed in the case of 4 mm thick parts.

By comparing the information that was obtained from the SEM observations, the cell density measurements, and cell size distributions, it is possible to deduce that the 3 mm thick parts that were molded with gas injection pressure 120 bar (GAS 2) have the largest cell density and the most homogeneous morphology, proved by the comparable cell size distribution between transition zone and core. In fact, the high cooling rate of the 2 mm thick parts freezes the polymer and it does not allow the growth of the cells. Vice versa, the lower cooling rate in the 4 mm thick part allows the growth of the cells and their coalescence.

### 3.2. Thermal Properties

**Figure 11:**
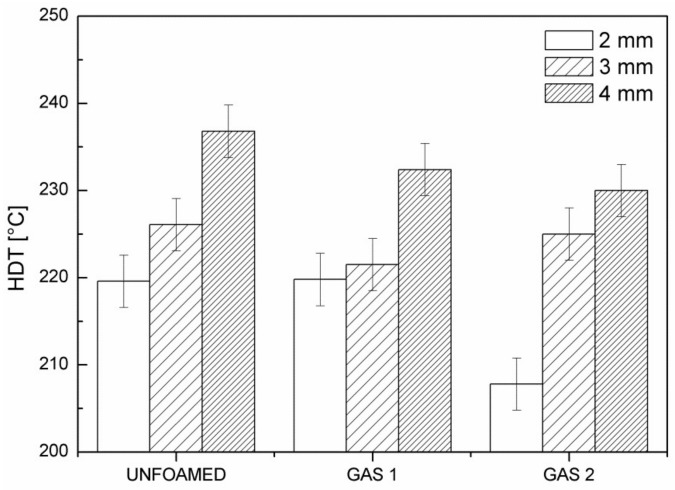
Heat Deflection Temperature for unfoamed samples, and GAS 1 and GAS 2 samples at all of the three part thicknesses.

Figure 11 reports the HDT of the foamed and unfoamed samples with the three analyzed part thicknesses.

As clear from the figure, the thermal properties of the foamed components considerably increase with increasing the part thickness, confirming the best performance of the 3 mm and 4 mm thick parts. Furthermore, it is possible to observe an increase in HDT with the foaming degree, probably because the better expansion of the gas improved the distribution of the fibers along the component. The glass fiber orientation distribution, in fact, is a crucial microstructural parameter that affect thermal and mechanical property enhancement of the molded part. It is known that, in a solid injection molded part, the glass fibers tend to arrange in a multilayer structure through the thickness of the mold cavity due to the influence of the material flow. In particular, in the skin and transition layers, the shear effect is more marked and glass fibers are consequently oriented in the melt flow direction, while in the core region the melt flow is subjected to the minimum shear and the orientation is considerably lower [32]. However, in the case of foam injection molding, the analysis of the glass fiber orientation distribution becomes more complex. In fact, a rearrangement of the glass fibers occurs during cell growth: glass fibers exhibit both rotational and translational displacements that are in close proximity to the growing cells due to the melt’s biaxial stretching. These movements are strictly dependent on the cell size, the initial cell-fiber distance, and the initial fiber position [33].

### 3.3. Mechanical Properties

**Figure 12:**
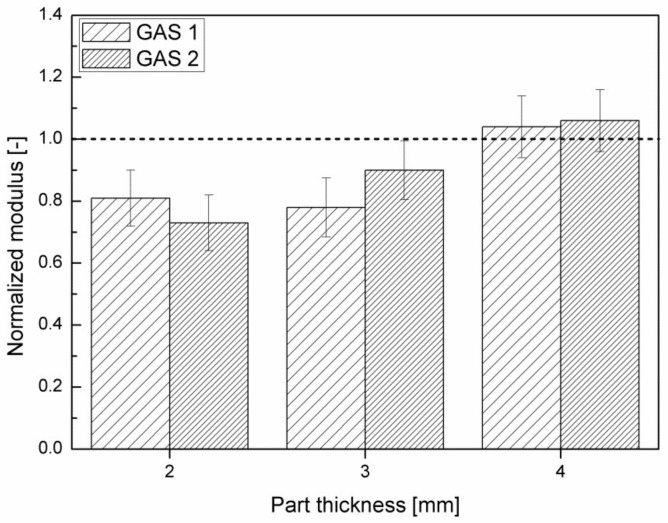
Normalized modulus of all the samples molded with injection temperature 300 °C.

Figure 12 shows the normalized modulus of all the samples molded with injection temperature 300 °C.

It is possible to note an increasing trend of the normalized modulus with the part thickness, until it reaches values close to 1 for the 4 mm thick samples. Values of normalized modulus that are equal to 1 mean that the reduction in modulus of the foamed parts with respect to the modulus of the corresponding compact parts is completely offset by the reduction in density. Furthermore, it is possible to observe a higher normalized modulus in the case of GAS 2 samples with respect to the GAS 1.

According to Equations (5) and (6), the flexural modulus of the structural foam is closely correlated to the core thickness and the core density. If the sample is assumed to be a system that is composed by two layers (Gonzalez approach), a foamed core, and a compact skin, the parameter *f* in equation 6 becomes equal to the density reduction that is given by Equation (3).

**Figure 13:**
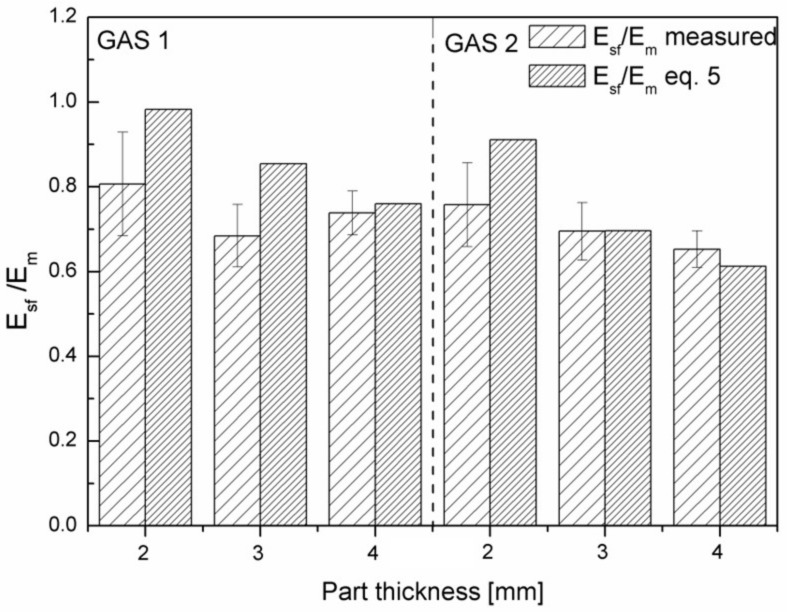
Ratio between flexural modulus of the structural foam and flexural modulus of the matrix: comparison of experimental values and values predicted by Equation (5).

On making this assumption, since the values of density reduction and of thickness of the core layer are known, Equation (5) can provide the values of the modulus of this sandwich structure. The calculation is reported in Figure 13.

It can be noticed that the expected modulus for the samples having a thickness of 2 mm is larger than the measured one. This indicates that the sandwich structure is not suitable in this case to describe the real structure of the sample. The same happens for the sample 3 mm GAS 1. The modulus is instead very well predicted for the samples that have a thickness of 4 mm or for the sample that is 3 mm thick with the largest gas quantity. For these samples, a sandwich structure describes the mechanical behaviour very well and equation 5 becomes predictive.

### 3.4. Analysis of Variance (ANOVA)

Table 5, Table 6 and Table 7 show the results of the ANOVA tests. A significant dependence of the part density on the part thickness and gas injection pressure (*p*-value < 0.0001) was found, with a percentage contribution of 56.46% for part thickness and 24.53% for gas injection pressure.

Part thickness is also a significant parameter for the flexural modulus (*p*-value = 0.0077), with a percentage contribution of 66.36%, while the injection temperature and gas injection pressure seem to have a significant influence on the cell density in the core (*p*-value = 0.0008), with a percentage contribution of 44.67% for gas injection pressure and 24.29% for injection temperature. The dependence can be quantified by estimating the coefficient in terms of coded factors, which represents the expected change in response y per unit change in x when all the remaining factors are held constant. Table 8 summarizes the obtained coefficients.

**Figure 14:**
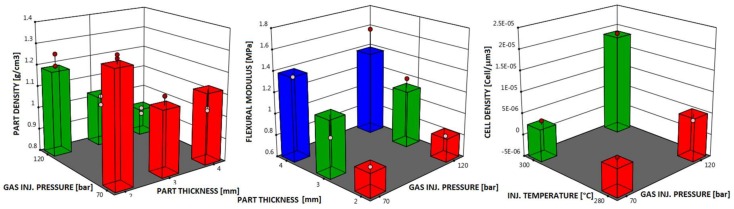
Factor coding of part density (values at 300 °C), flexural modulus (values at 300 °C), and cell density in the core (values at 4 mm). Bars represent the predicted values, points represent the values below (white) and above (red) the predicted values.

Figure 14 shows the values predicted by the analysis and their difference as compared to the experimental points.

The analysis of variance leads to the conclusion that the part density is affected by the part thickness and the gas injection pressure: low density can be obtained with high gas injection pressure and high part thickness. Flexural modulus is prevalently dependent on the part thickness: by increasing the part thickness, the flexural modulus increases. Injection temperature and gas injection pressure are significant parameters for cell density in the core: in order to have high cell density, high temperature and gas pressure should be used. In summary, in order to obtain homogeneous foamed parts with good flexural modulus, an injection temperature of 300 °C, gas injection pressure of 120 bar, and a thickness of 4 mm should be adopted.

## 4. Conclusions

In this work, the influence of some processing parameters, such as injection temperature, gas injection pressure, and part thickness on the morphology of PA66 + 30% glass fiber foamed parts, on their density, thermal, and mechanical performances was investigated. Microcellular injection molding technology was successfully employed to produce glass-fiber-reinforced PA66 composites with tailored sandwich microstructure (compact skin, foamed core). The injection temperature resulted in strongly influencing the cell density in the core layer, as demonstrated by SEM analysis and the ANOVA test. In fact, the higher viscosity of the thermoplastic material at 280 °C did not allow for good mixing between the gas and the polymer. The samples that were molded with an injection temperature of 300 °C displayed weight and density reduction increases with the gas injection pressure and with the part thickness. In particular, samples with a part thickness of 4 mm foamed with the highest gas injection pressure reached 25% weight reduction and a density reduction corresponding to 34%. A remarkable improvement in performance with the increasing of gas injection pressure was found. In particular, at high gas injection pressures, a better foamed core layer and higher mechanical properties was observed. Mechanical properties also resulted in being strongly influenced by the morphology distribution in the sandwich structure: by increasing the core thickness and its density, the flexural modulus decreased. All experimental analysis led to the conclusion that the core morphology is crucial to the properties of a molded part. According to ANOVA results, in order to obtain homogeneous foamed parts with good mechanical properties, an injection temperature of 300 °C, gas injection pressure 120 bar, and large thick parts should be adopted. The thermal properties of the foamed components considerably increased on increasing the part thickness, confirming the best performance of the 3 mm and 4 mm thick parts.

## Figures and Tables

**Table 1:** Parameters of Cross-WLF Model.

Parameter	CASE 1
**n**	0.2
τ*	5500 Pa
**D1**	3.00 × 10^30^ Pa·s
T*	323.17 K
**A1**	76.1
**A2**	51.6 K

**Table 2:** Processing conditions.

Process Parameter	CASE 1	CASE 2
Injection temperature (°C)	300	280
Mold temperature (°C)	90	90
Injection Flow Rate (cm^3^/s)	26	26
Screw rotation during dosage (rpm)	200	200
Back pressure (bar)	0	0
Max filling pressure (bar)	70	70
Gas injection pressure (bar)	70–120	70–120
Cavity thickness (mm)	2–3–4	2–3–4

**Table 3:** Amount of gas in grams on injection volume corresponding to the gas injection pressures.

Condition	Gas Injection Pressure (bar)	Gas Amount/Injection Volume (g/ccm)		
		2 mm	3 mm	4 mm
GAS 1	70	0.020	0.013	0.013
GAS 2	120	0.038	0.036	0.031

**Table 4:** Skin thickness.

Condition	Skin Thickness (μm)		
	2 mm	3 mm	4 mm
GAS 1	200	280	180
GAS 2	280	190	170

**Table 5:** Analysis of variance (ANOVA) results on part density.

Source	Sum of Squares	df	Mean Square	F-value	*p*-value	Dependence	Percentage Contribution
Model	0.3559	5	0.0712	19.79	<0.0001	significant	-
Part thickness (A)	0.2355	2	0.1177	32.74	<0.0001	-	56.46%
Gas injection pressure (B)	0.1023	1	0.1023	28.46	<0.0001	-	24.53%
AB	0.0181	2	0.0091	2.52	0.1100	-	4.34%
Residual	0.0611	17	0.0036	-	-	-	14.65%
Cor Total	0.4171	23	-	-	-		-

**Table 6:** ANOVA results on normalized modulus.

Source	Sum of Squares	df	Mean Square	F-value	*p*-value	Dependence	Percentage Contribution
Model	0.6636	2	0.3318	8.78	0.0077	significant	-
Part thickness	0.6636	2	0.3318	8.78	0.0077	-	66.36%
Residual	0.3400	9	0.0378	-	-	-	34.00%
Cor Total	1.00	11	-	-	-	-	-

**Table 7:** ANOVA results on cell density in the core layer.

Source	Sum of Squares	df	Mean Square	F-value	*p*-value	Dependence	Percentage Contribution
Model	5.805 × 10^−10^	3	1.935 × 10^−10^	16.86	0.0008	significant	-
Gas Injection pressure (A)	3.003 × 10^−10^	1	3.003 × 10^−10^	26.17	0.0009	-	44.67%
Injection temperature (B)	1.633 × 10^−10^	1	1.633 × 10^−10^	14.23	0.0054	-	24.29%
AB	1.168 × 10^−10^	1	1.168 × 10^−10^	10.18	0.0128	-	17.37%
Residual	9.180 × 10^−10^	8	1.148 × 10^−11^	-	-	-	13.65%
Cor Total	6.723 × 10^−10^	11	-	-	-	-	v

**Table 8:** ANOVA coefficients Table.

Parameter	Intercept	Factor A: Injection Temperature	Factor B(1): Part Thickness	Factor B(2): Part Thickness	Factor C: Gas Injection Pressures	Factor AC	Factor B(1)C	Factor B(2)C
Part density	1.115293	-	0.1381	−0.048607	−0.0653	-	0.0038	0.0316
*p*-value	-	-	<0.0001	<0.0001	<0.0001	-	0.1100	0.1100
Normalized modulus	1.12	-	−0.2975	0.02	-	-	-	-
*p*-value	-	-	0.0077	0.0077	-	-	-	-
Cell density	7.09 × 10^−6^	3.69 × 10^−6^	-	-	5.00 × 10^−6^	3.12 × 10^−6^	-	-
*p*-value	-	0.0054	-	-	0.0009	0.0128	-	-
